# Red Wood Ants Display Natural Aversive Learning Differently Depending on Their Task Specialization

**DOI:** 10.3389/fpsyg.2019.00710

**Published:** 2019-03-29

**Authors:** Ivan Iakovlev, Zhanna Reznikova

**Affiliations:** ^1^Institute of Systematics and Ecology of Animals, Siberian Branch of the Russian Academy of Sciences, Novosibirsk, Russia; ^2^V. Zelman Institute for Medicine and Psychology, Novosibirsk State University, Novosibirsk, Russia

**Keywords:** ants, specialization, task, syrphid, aversive learning, aggressive behavior

## Abstract

The adaptive benefits of individual specialization and how learning abilities correlate with task performance are still far from being well-understood. Red wood ants are characterized by their huge colonies and deep professional specialization. We hypothesized that red wood ants *Formica aquilonia* form aversive learning after having negative encounters with hoverfly larvae differently, depending on their task specialization. We tested this hypothesis, first, by examining whether hunters and aphid milkers learn differently to avoid the nuisance of contacts with syrphid larvae, and, second, by analyzing the difference between learning in “field” and laboratory-reared (naïve) foragers. During the first interaction with the syrphid larva in their lives the naïve foragers showed a significantly higher level of aggressiveness than the members of a natural colony. Naïve foragers applied the “mortal grip,” “prolonged bites,” and “nibbling” toward the enemy with a significantly higher frequency, whereas members of both “field” groups behaved more carefully and tried to avoid encounters with the larva. The aphid milkers, who had a negative experience of interaction with the larva, being “glued” with its viscous secretion, behaved much less aggressively in the follow-up experiments after 10 min and even 3 days, thus exhibiting the shaping of both short- and long-term memories. However, both “field” hunters and naïve foragers demonstrated no signs of aversive learning. These data provide some new insights into the relationship between task specialization and learning performance in ants. Given our previous results, we speculate that scouts and aphid milkers are the most cognitively gifted specialists in red wood ants, whereas hunters and guards are rather brave than smart.

## Introduction

Eusocial insect species form persistent colonies, where groups of individuals (castes) perform different tasks (division of labor). Not only members of the reproductive castes, such as queens, have clearly defined responsibilities with workers, but the workers themselves also have particular tasks such as caring for the young, defense, foraging, nest-building activities, and so on (for a review see: Robinson, 1992). The main characteristic of insect societies is the way tasks are distributed among group members. The adaptive benefits of individual specialization and how learning abilities correlate with task performance are still far from being understood. Ants are good candidates for studying these problems as a highly diverse and successful group of social hymenopterans, which, unlike bees and wasps, consists only of eusocial species (for a review see: Reznikova, 2018). There are more than 12,000 ant species in the world, with different colony sizes (from tens to millions of individuals), social life and styles of cooperation, from single foraging to mass recruiting. Ant species display different modes of the division of labor and the specialization of workers on various tasks. Caste polyphenism is rather expressive in some ant species, which harbor special sub-castes of workers profoundly different by morphology, physiology, and behavior (for a review see: Hölldobler and Wilson, 1990). In leaf-cutting ants, for instance, tiny “mini workers” cultivate fungi in the subterranean nest to feed the larvae. Other workers have an up to 200-fold increased body weight, leaving the nest for long foraging trips, and bringing back leaves which are used as substrate for the fungi (Wilson, 1980). However, a morphological caste system may also have costs, as it may prevent a colony from rapidly adjusting caste ratios, increase the energetic cost of rearing or limit the task repertoire (Oster and Wilson, 1978). In the majority of ant species, workers only specialize in different tasks behaviorally. In some ant species, behavioral specialization is strongly determined by chronological age and physiological development (temporal polyethism): young workers typically perform safe tasks inside the nest, such as nursing the brood, and only later in life move on to more risky tasks outside the nest, such as foraging or territorial defense (Hölldobler and Wilson, 1990; Beshers et al., 2001; Giraldo and Traniello, 2014). In other species, task specialization among workers may be shaped by their size polymorphism, genetic background, experience, and social interactions, which is also partly influenced by age (Tripet and Nonacs, 2004; Schwander et al., 2005; Ravary et al., 2007; Helanterä et al., 2013; Giehr et al., 2017; Doering et al., 2018). Behavioral and cognitive mechanisms of the specialization of workers on different tasks remain the least studied in this area.

Red wood ants (the *Formica rufa* group) are possibly the most promising group for studying the role of learning and experience in task specialization among workers (Reznikova, 2018). In comparison with many sympatric species, the mound-building red wood ants have hundreds of times more individuals in their colonies and spacious feeding territories (Dlussky, 1967; Rosengren and Sundström, 1987). Every day red wood ants face complex vital problems: for example, in order to obtain honeydew, the basic food for adults, thousands of colony members have to find and memorize locations of a large number of aphid colonies within such a huge three-dimensional space as a tree is for an ant (Reznikova, 2008). Dobrzanska (1958) demonstrated that in red wood ants, groups of individuals return repeatedly to approximately the same parts of the colony’s feeding territory on the ground and work together there. Studying site allegiance in red wood ants, Rosengren and Fortelius (1987) characterized red wood ants as “replete ants” storing not lipids in their fat bodies but habitat information in their brains, and members of this group of species used to be a relevant model for studying spatial learning and intelligence (Reznikova and Ryabko, 1994; Nicholson et al., 1999).

It was demonstrated in early studies that in red wood ants, out-nest workers include hunters and collectors of nest material acting on the ground, aphid milkers collecting honeydew on the trees and hunters collecting prey there, as well as guards defending the nest entrances (Otto, 1958; Horstmann, 1973). Studies at the individual level revealed deep professional specialization, that is, considerable behavioral differences between members within different task groups. For instance, the task group of aphid tenders turned out to include professional subgroups such as scouts, aphid milkers (“shepherds”), aphid guards, and carriers (Reznikova and Novgorodova, 1998a; Reznikova, 2007). Experimental studies of interactions of the red wood ant *Formica aquilonia* with ground beetles, their eternal enemies, large and dangerous, revealed that nest guards, and hunters are much more aggressive than aphid milkers (Reznikova and Dorosheva, 2004; Dorosheva et al., 2011). Experiments with other intruders, such as spiders and the small parasitic rove beetle, revealed a context-dependent specialization in colony defense in the red wood ant *F. rufa*: small workers were better at preventing brood predation than larger workers, and nurses and workers at nest entrances were more aggressive toward parasitic beetles than extranidal foragers (Parmentier et al., 2015).

Reznikova and Ryabko (1994, 2011) revealed in red wood ants a group-retrieving mode of foraging basing on the difference of the searching activity and cognitive abilities between scouts and recruited foragers. Scouts appeared to be able to grasp regularities in the sequences of turns (right and left) in the “binary tree” maze on the way to the goal, and use them to optimize their messages to recruited foragers, whereas the recruited foragers can only memorize and not transfer the information. The sophisticated communication system between the scouts and the recruited foragers is even more complicated than the honeybee dance language (see details in: Reznikova, 2017). Experiments with a battery of behavioral tests demonstrated that scouts form spatial memory faster and keep information longer than recruited foragers. They were, in general, more exploratory than other out-nest workers, more readily switched between different activities in unfamiliar situations, and, although displaying an intermediate level of aggressiveness between aphid milkers and nest guards, they never attacked the enemy directly (Atsarkina et al., 2014; Reznikova, 2018).

A question then arises about the distribution of cognitive responsibilities within the ant colony (sensu: Reznikova, 2008), that is, about the differentiation between groups in their abilities to perform cognitively demanding tasks. In this study, we concentrate on how representatives of different task groups shape natural aversive learning in the context of repeated interactions with an enemy. Similar with Hollis et al. (2017) study, we use the term “natural aversive learning” meaning ants’ behavior toward their natural enemies or/and predators, although agreeing with Hénaut et al. (2014) that even if a species demonstrates aversive learning in the laboratory, most often it is difficult to determine whether such a capacity is likely to occur in natural conditions (see also: Bernays, 1993).

Aversive learning has been studied in ants mainly on the basis of one-trial learning. Ants’ memory for an aversive event was tested shortly after their unpleasant experience. For example, *Ectatomma tuberculatum* appeared to learn how to break quickly through the spiders’ web and kept this memory during 15 min after the first experience (Hénaut et al., 2014), *Formica pratensis* retained the memory of a single unpleasant collision with a hoverfly larva for 10–30 min after the event (Novgorodova, 2015), and pavement *Tetramorium* ants learned to avoid antlion traps following a single successful escape from a pit for 1 min after the encounter (Hollis et al., 2017). Long-term memory for an aversive event was demonstrated in the experiments of Dejean (1988) with *Odontomachus troglodytes*. These ants kept the memory about encounters with the chemically defensive larva of the African chrysomeline during 28 days. As far as we know, natural aversive learning in ants has never been considered in the context of task specialization.

The interaction between red wood ants and hoverfly larvae can serve as a natural and somehow unique experimental model in studying differences in learning abilities between members of different task groups. Hoverfly (Diptera, Syrphidae) females use semiochemicals to locate aphid colonies and to oviposit eggs from which aphidophagous larvae hatch (see reviews in: Detrain et al., 2017). It is known that aphids provide a vital energy source that is essential for the survival and growth of the ant colony (Addicott, 1978; Skinner and Whittaker, 1981), and ants actively protect their trophobionts from all aphidophages (Novgorodova and Gavrilyuk, 2012). Hoverfly larvae use adhesive saliva to incapacitate an attacker (Eisner, 1972; Rotheray, 1986). Ecological aspects of intricate interplay between ants and hoverfly larvae have been studied recently on *F. pratensis* by Novgorodova (2015) and on *Lasius niger* by Detrain et al. (2017). Both studies showed that to prevent predation of aphids by hoverfly larvae, ants demonstrated aggressive behavior; once bitten by ants, third instar hoverfly larvae released a droplet of viscous and sticky secretion from the mouth, which hardens like glue. These actions make ants stop their attacks to clean themselves and decrease aggression toward the larvae for a few minutes. Since the impact of the larvae on ants is not dangerous, but only unpleasant for them, ants can interact with the enemy many times in a row. This experimental model allows one to check if ants are learning to avoid unpleasant encounters.

We, therefore, hypothesized that the members of different task groups form aversive learning after having negative encounters with hoverfly larvae differently depending on their tasks. We tested this hypothesis, first, by examining whether hunters and aphid milkers from the natural colony of *F. aquilonia* learn differently to avoid the nuisance of contacts with hoverfly larvae, and, second, by analyzing the difference between learning in the “field” and naïve foragers. We successfully showed that the aphid milkers shaped both short- and long-term memories about a negative experience of interaction with the enemy, whereas hunters and naïve foragers did not change their behavior after the unpleasant event. As far as we know, this is the first demonstration of natural aversive learning in ants in the context of task specialization.

## Materials and Methods

### Insects

We collected ants near Novosibirsk, western Siberia, Russia (54 50.1N, 83 06.1E), in a mixed birch-pine forest. Hunters and aphid milkers were taken from the colony of *F. aquilonia.* We regarded as hunters those ants which carried insect prey on foraging trails toward the nest. The aphid milkers were collected on colonies of *Symydobius oblongus* aphids on *Betula pendula* where ants tend the aphids and obtain honeydew from them. These “field” hunters and aphid milkers were kept in the laboratory, in separate artificial nests located in separate arenas (40 cm × 20 cm × 20 cm) in groups of 100 individuals. We formed the naïve colony from the ants hatched in the laboratory from pupae taken from the same colony of *F. aquilonia.* The naïve colony included about 800 workers, brood and the queen, and was kept in an artificial nest located in an arena measuring 150 cm × 80 cm × 20 cm during more than a year, without any contacts with the “field” workers or other insects. For the experiments, we selected naïve workers 13–14 months of age that were engaged in collecting both carbohydrate and protein food as well as the nest material in the arena and carrying ant corpses out of the nest. Similar to the study of Reznikova and Novgorodova (1998b) conducted on naïve ants earlier, there were no distinguishable task groups among foraging naïve workers, which is why we regarded them merely as “naïve foragers.” All ants obtained sugar syrup as the carbohydrate food and crumbled eggs as the protein food. Members of all groups were individually marked, which helped us to distinguish them in the follow-up experiments. Paints of 6 colors and a five-point code were used: one mark on the head, two on the thorax, and two on the abdomen. Tags were renewed every 1–2 weeks. Hoverfly larvae were collected in mixed forests near Novosibirsk from various plants and kept individually in aerated plastic boxes (10 cm × 6 cm × 4 cm), with plants infested with aphids.

### Behavior of Ants and Hoverfly Larvae in Dyadic Interactions

To record and describe the behavior of the insects, we use the notions “behavioral element” and “behavioral pattern” as it is used in Reznikova et al., 2017, that is, referring to a certain behavioral act (such as “bite”), and a certain sequence of elements (such as “hunting”).

To compare the agonistic behavior toward an enemy in different groups of ants (“field” aphid milkers and hunters, and naïve foragers), we observed pairwise interactions between ants and hoverfly larvae in plastic containers (20 cm × 8 cm × 4 cm) covered with glass. Each test lasted 10 min after the first contact between the insects. Similar with the Detrain et al. (2017) study, we used the third instar syrphid larvae of 9–13 mm length. We tested 36 aphid milkers, 20 hunters, and 16 naïve foragers. It is worth to note that, in contrast with the interaction with dead prey, when the behavior of red wood ants depends on the size of the group (Szczuka and Godziñska, 2004), they behave very actively in pairwise interaction with living enemies and competitors (Reznikova and Dorosheva, 2004).

We recorded ten elements of agonistic behavior of ants (ordered here by the degree of aggressiveness): (i) “avoidance”: running away; (ii) “ignoring”: an ant comes close to a larva and does not pay any attention to it; (iii/iv) “antennation”: investigation of a larva by antennae with open/closed mandibles; (v) “hit-and-run attack”: a sudden lunge toward a larva with open mandibles followed by a motion backward; (vi) “nibbling”: short series of touching the enemy with open mandibles; (vii) “short bites” (lasting 1–5 s); (viii) “prolonged bites” (more than 5 s); (ix) “mortal grip”: prolonged capturing of the larva using mandibles and legs usually accompanied by spraying the larva with acid; (x) “transporting”: the ant carries the larva in its mandibles and runs around. We also recorded self-grooming behavior in ants, that is, the behavior of cleaning the body, legs, and antennae. We split behavioral elements into two groups: “non-aggressive” (i–iv) and “aggressive” (v–x).

Two behavioral elements of the target hoverfly larvae were recorded: (i) “freezing” motionless and (ii) “defensive flexion of the anterior body part” where the larva bent its head toward the attacking ant. The last element could be accompanied with a releasing of defensive adhesive secretion from the mouth which hardens like glue. Being glued by a larva, an ant stops its attack and has to clean itself for a time.

### Studying Ants’ Learning Abilities to Avoid Unpleasant Encounters With an Enemy

We studied short-term and long-term learning abilities of ants to avoid the nuisance of contacts with the chemically defensive enemy. To examine short-term learning (less than 10 min after gaining negative experience), we compared two ants’ behaviors toward a hoverfly larva during the first tests before and after the larvae glued the ant. Sometimes the larva glued the ant 2–3 times during the first test; in this case, we considered only the behavior of the ant after the first “gluing.”

To examine long-term learning, we conducted the follow-up experiment with an interval of 1–3 days. To show that the training of different groups took place in a similar mode, we checked the differences between the groups by inter-test intervals. No significant differences in the inter-test intervals between ant groups were observed: ANOVA, *F*_2,25_ = 0.309, *p* = 0.737; means ± SEM: 1.8 ± 0.3 days for aphid milkers, 2.0 ± 0.4 days for hunters, 1.7 ± 0.3 days for naïve foragers. Thus, we can compare the ability for long-term learning between groups of ants. Out of the ants that were glued by syrphids and thus obtained negative experience in the first test, we selected for the follow-up experiment nine aphid milkers, seven hunters, and twelve naïve foragers. Each syrphid larva was used only once. Test containers were washed with ethanol after each test. In total 100 tests were conducted, and the whole time of observation made up about 17 h.

### Data Analysis

We measured ants’ behavior during contacts with the enemy assigning each behavior a score according to the following order of aggressiveness: 1 – avoidance, 2 – ignoring, 3 – antennation, 4 – short attack (hit-and-run attack, nibbling, and short bites), 5 – prolonged attack (prolonged bites, mortal grip, and transporting). For each test, the level of ants’ aggressiveness was calculated as the sum of the scores of all behaviors of the ant divided by the total number of ant-syrphid contacts during the test. If an ant demonstrated a range of behavioral elements, quickly changing from one to another during its contact with the larva, we analyzed the most aggressive elements only. When analyzing short-term learning, aggression scores were counted only for two encounters, before, and after the larva glued the and for the first time, that is, we considered only the very first chemical defensive reaction of the larvae. We calculated the occurrence of distinct behavioral elements for each ant group as the percentage of ants that demonstrated a distinct behavioral element toward hoverfly larvae from the total number of ants tested.

Since the data were normally distributed (Kolmogorov-Smirnov test, *p* > 0.05), we used parametric tests. Comparison of the levels of aggressiveness between groups of ants was performed using univariate analysis of variance (one-way ANOVA, Tukey HSD *post hoc* test). The significance of differences in the occurrence of behavioral elements was determined using Fisher’s exact test. To compare the behavior of the same ants at different stages of the experiment, the *t*-test for paired samples was used. All results were analyzed using Microsoft Excel and SPSS v.22.

## Results

### Behavior of Ants and Hoverfly Larvae in Dyadic Interactions

**FIGURE 1:**
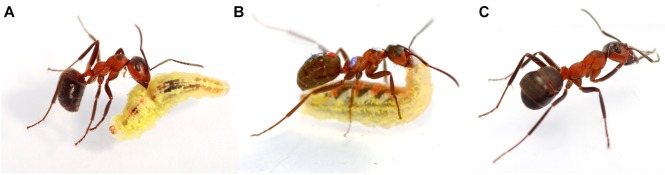
The behavior of red wood ants and hoverfly larvae in pairwise interactions. **(A)** Ant bites the enemy. **(B)** Hoverfly larva shows active defense by releasing a droplet of viscous and sticky secretion from the mouth, which hardens like glue. **(C)** Ant stops attacking to clean itself.

During their encounters with ants, hoverfly larvae demonstrated freezing and active chemical defense, including flexion of their anterior body part toward the attacking ant and releasing a sticky secretion over it (Figure 1A–C). The behavior of syrphids depended on the aggressiveness of the ants. In 54 of the 67 encounters, the larvae demonstrated the defensive behavior in response to the following aggressive behaviors of ants: bites (39 out of 67), nibbling (12 out of 67), and hit-and-run attacks (3 out of 67) (Figure 1A). After being touched by the mouthparts of the syrphid and getting on the body of syrphids’ defensive secrets (Figure 1B), the ants walked in a jerky or staggering way, rubbing their mandibles over the ground, and thoroughly cleaned their antennae (Figure 1C). In the first test, the syrphid larvae “glued” a similar portion of “field” ants in each task group, 17 out of 36 aphid milkers and 11 out of 20 hunters (Fisher’s exact test, *p* > 0.05), and significantly higher portion of naïve foragers (12 out of 16) relative to “field” aphid milkers (Fisher’s exact test, *p* < 0.05).

**FIGURE 2:**
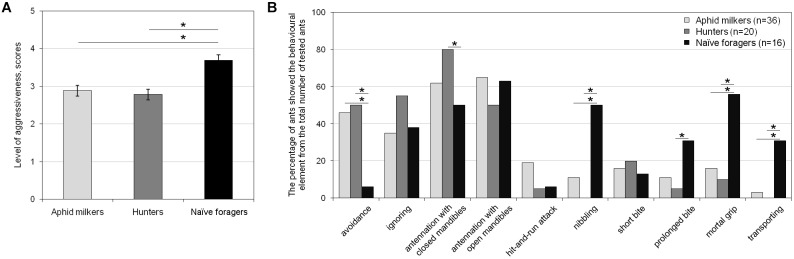
Differences in **(A)** the level of aggressiveness and **(B)** the occurrence of behavioral elements between aphid milkers, hunters, and naïve foragers during their interactions with hoverfly larvae in the first test. Significant differences are marked with asterisks: **(A)** mean ± SEM, one-way ANOVA, *p* < 0.01, Tukey HSD, *p* < 0.01; **(B)** Fisher’s exact test, *p* < 0.05.

We compared the level of aggressiveness and occurrence of behavioral elements in aphid milkers, hunters, and naïve foragers. First, we found an essential difference between both groups of “field” ants and the naïve foragers during the first encounters with syrphids (Figure 2). During the first interaction with this enemy in their lives, naïve foragers showed a significantly higher level of aggressiveness than the members of a field colony (*F*_2,70_ = 7.03, *p* = 0.002, Tukey HSD, *p* = 0.003; Figure 2A). Naïve foragers applied the mortal grip and nibbling toward the enemy with a significantly higher frequency, whereas members of both “field” groups tried to avoid encounters with the larvae (Figure 2B). In comparison with “field” hunters, naïve foragers applied prolonged bites much more often, and less frequently they investigated the larvae with their antennae, keeping mandibles closed (Figure 2B). It is worth to note that the transition from the prolonged attack to transporting a syrphid larva occurred much more frequently in the naïve foragers (Figure 2B). Of the seven naïve foragers who applied a mortal grip on the larva, five ants bore it, and two of them killed the enemy. Both “field” aphid milkers and hunters showed similar levels of aggressiveness (*F*_2,70_ = 7.03, *p* = 0.002, Tukey HSD, *p* > 0.05; Figure 2A) and a similar occurrence of behavioral elements (Figure 2B). Most of the “field” ants showed non-aggressive behaviors toward syrphids (such as avoidance, ignoring, and antennation), and no more than 20% of both aphid milkers and hunters showed attack behaviors such as hit-and-run attacks, nibbling, bites, and the mortal grip (Figure 2B).

### Short-Term Aversive Learning in Ants

**FIGURE 3:**
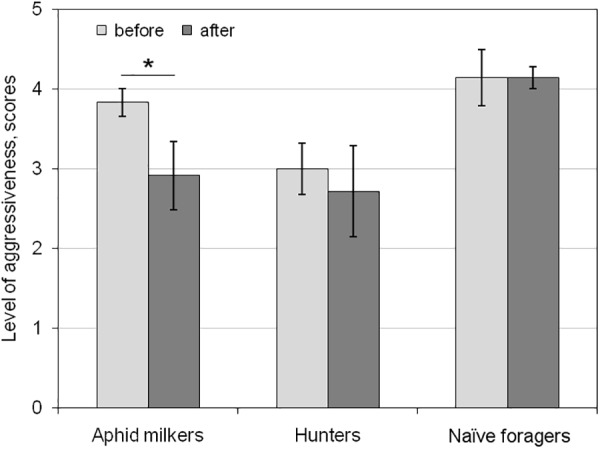
Short-term changes in the level of aggressiveness in aphid milkers, hunters, and naïve foragers before and after the hoverfly larva glued the ant during the first test. Significant differences are marked with asterisks (mean ± SEM; *t*-test for paired samples, *p* < 0.05).

To examine the short-term aversive learning in ants, we compared two behaviors of ants toward syrphid larvae during the 10-min test: before and after the first gluing the ant by the larva. After cleaning of larva’s secretion, the aphid milkers reacted to the enemy much less aggressively (t(11) = 2.561, *p* < 0.05; Figure 3). The hunters and naïve foragers did not change their aggressiveness after cleaning of larva’s secretion (t(6) = 0.42 and t(6) = 0, correspondingly, *p* > 0.05; Figure 3).

### Long-Term Aversive Learning in Ants

**FIGURE 4:**
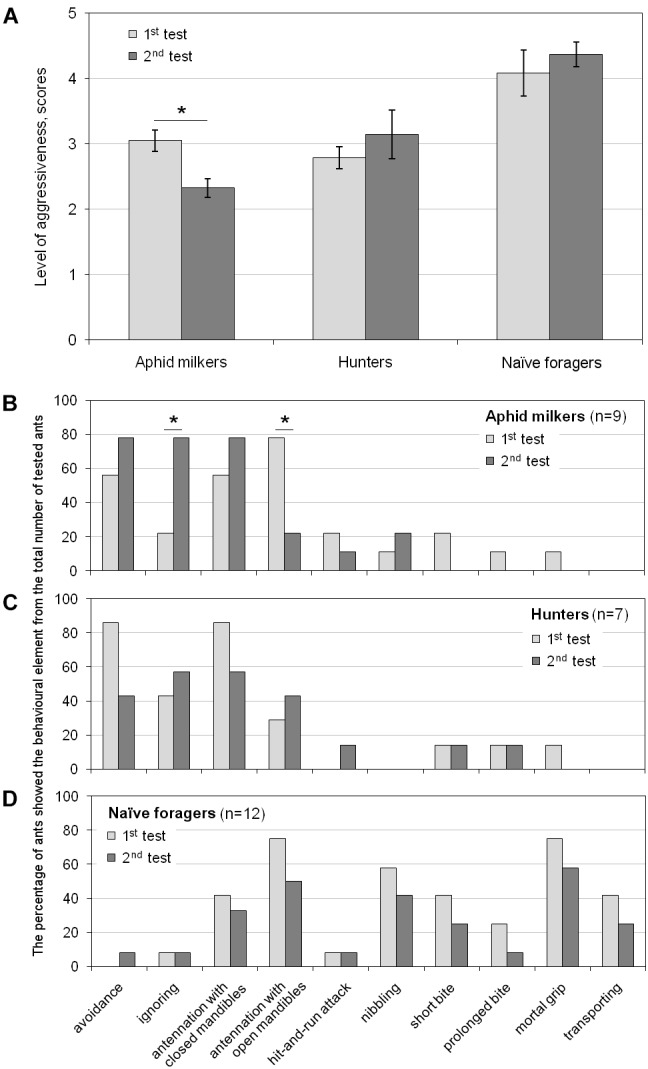
Long-term changes in **(A)** the level of aggressiveness and the occurrence of behavioral elements in **(B)** aphid milkers, **(C)** hunters, and **(D)** naïve foragers between the first and the follow-up tests. Significant differences are marked with asterisks: **(A)** mean ± SEM, *t*-test for paired samples, *p* < 0.05; **(B–D)** Fisher’s exact test, *p* < 0.05.

To examine the shaping of long-term memory in ants about the nuisance of encounters with hoverfly larvae releasing droplets of viscous and sticky secretion, we conducted follow-up experiments after 1–3 days since the first one. The aphid milkers, who had a negative experience of interaction with the larva in the first test, behaved much less aggressively in the second test (t(8) = 2.943, p < 0.05; Figure 4A). The occurrence of “ignoring” increased in their behavioral patterns, and the occurrence of “antennation” decreased (Figure 4B). In contrast to the aphid milkers, the hunters and the naïve foragers, who had a negative experience of interaction with the larva in the first test, showed the same level of aggressiveness in the second test (t(6) = -0.956 and t(11) = -1.245, correspondingly, *p* > 0.05; Figure 4A). Both hunters and naïve foragers demonstrated a similar occurrence of different behavioral elements during the first and the follow-up experiments (Figure 4C,D).

## Discussion

In our experiments with *F. aquilonia*, during the first interaction with the syrphid larva in their lives, the naïve foragers showed a significantly higher level of aggressiveness than the members of a natural colony. Naïve foragers applied the mortal grip, prolonged bites, and nibbling toward the enemy with significantly more frequency, whereas members of both “field” groups behaved more carefully and tried to avoid encounters with the larva. The aphid milkers, who had a negative experience of interaction with the larva, being “glued” with its viscous secretion, behaved much less aggressively in the follow-up experiments after 10 min and even 3 days, thus shaping both short- and long-term memories. However, both “field” hunters and naïve foragers demonstrated no signs of aversive learning. As far as we know, this study provides the first link between the natural aversive learning performance and task specialization in ants. A question then arises, what behavioral and cognitive traits of the members of different task groups are responsible for the differences in their abilities to form natural aversive learning.

First, the difference in the previous experience could influence the abilities to form natural aversive learning in the hunters and the aphid milkers in our experiments with hoverfly larvae. It has been recently demonstrated that in the red wood ant *F. rufa* workers inside the nest interact more frequently with the myrmecophile parasites than foragers, and prior encounter and greater experience in attacking these parasites could cause nurses and mound workers to recognize them more rapidly as a threat than foragers would do and to have a lower threshold to initiate aggression (Parmentier et al., 2015). In our experiments, aphid milkers taken from aphid colonies on a tree were more likely to have experience of previous encounters with the hoverfly larvae and other aphidophages such as ladybird larvae, all chemically defensive, whereas land hunters were unlikely to encounter these insects, and naïve foragers completely lacked the previous experience of interaction with chemically defensive insects. This assumption, although indirect, is also confirmed by differences in the behavior of hunters and naïve foragers toward the enemy. In our experiments, naïve foragers of 13–14 months of age, applied much more aggressive reactions toward the hoverfly larvae than the members of both “field” groups. During the follow-up experiments, naïve foragers behaved less carefully than “field” hunters. Not only did they not learn to avoid the syrphids, but in some cases, they passed on to predatory behavior toward these insects. Since syrphid larvae may occasionally be part of the ant diet (Punttila et al., 2004), naïve foragers can recognize them as “a general image of a victim” such as many larvae of other insects permanently found in the ants’ prey (Iakovlev et al., 2017), rather than “an enemy image.” The hoverfy larva, with its relatively safe gluing secretion, is much less dangerous than, say, predatory ground beetles who can kill the ants, and also have chemical protection. It has been demonstrated earlier that red wood ants possess an innate template for perception and identification of an “enemy image” including such features of the predatory ground beetles as dark coloration, the size, the presence of “outgrowths” (legs, antennae), body symmetry, the rate of movement, and scent (Dorosheva et al., 2011; Reznikova and Dorosheva, 2013). However, the ability to single out the key features and complete the integral image seems to require accumulation of experience (Reznikova and Iakovlev, 2008), and hunters are much more cautious toward the ground beetles than both nest guards and naïve workers (Iakovlev, 2010). In the present study, “field” hunters, although did not learn to avoid the negative encounters with hoverfly larvae, behaved more careful than naïve foragers, perhaps based on their experience of collisions with some chemically defensive insects on the soil surface.

The second reason explaining differences in the behavior of representatives of different ants’ task groups may be the manifestation of a set of task-specific behavioral features. It was demonstrated earlier that in red wood ants members of each task group possess a stable set of distinct behavioral characteristics (Reznikova, 2011). Experiments of Iakovlev (2010) with the battery of behavioral tests, in which ants interacted with artificial models of natural objects as well as with live predatory beetles, revealed particular suits of behavioral features of the members of different task groups. For instance, aphid milkers display a high level of exploratory activity with the preference for artificial grass stems, low aggressiveness, and evasion of contacts with the predators. At the sight of a stuffed blue tit, guards react aggressively and try to bite, whereas aphid milkers continue milking aphids or jump from the branch down (Iakovlev, 2010; Reznikova, 2011, 2017). Hunters display much more agility and aggressiveness than aphid milkers, and the low level of exploration activity (Iakovlev, 2010). There are similarities in behavioral traits of hunters and nest guards; however, the hunters are more careful, and in contrast to the nest guards, they never use the most dangerous methods of dealing with predatory beetles, such as mortal grip and long bites (Reznikova and Iakovlev, 2008). Similar correlations between behavior and specialization were revealed in other ant species. In *Myrmica rubra* colonies patrollers are bolder, more aggressive and more active than foragers and brood carers (Chapman et al., 2011). In *Camponotus* *aethiops*, the exploratory activity of workers in the open field significantly predict learning performance: “active-explorers” were slower in appetitive olfactory learning than “inactive-explorers,” and they are also more aggressive (Udino et al., 2017). In the present experiments, the ability of aphid milkers to learn how to avoid hoverfly larva can be associated with their low aggressiveness and conflict avoidance. It is likely that battles with aphidophages are left to the aphid guards who belong to the same aphid tending group and possess similar behavioral traits with the nest guards (Reznikova and Novgorodova, 1998a).

In sum, previous experience, innate level of aggressiveness and exploration, and templates of vital attention objects, together shape the difference in learning between representatives of different task groups. It is difficult, if not impossible, to separate the task specialization from the experience gained. Naive foragers in our experiments can serve as an example. Lacking experience with natural enemies, competitors and symbionts, having only vague templates of food and enemy images, they applied highly aggressive reactions toward dangerous animals, and did not learn how to avoid encounters. Keeping in mind the high cognitive abilities of scouts as the most intelligent task group in red wood ants’ colony described earlier (Reznikova and Ryabko, 2011), one can suggest that individual variation in aggressiveness, peculiarities of exploratory activity, orientation, learning, and memory underlies the specialization of workers in performing various tasks. Given our previous results, we speculate that scouts, and aphid milkers are the most cognitively gifted task groups in red wood ants, whereas hunters and guards are rather brave than smart.

There is much work to be done to evaluate the fitness consequences of deep professional specialization in red wood ants. The role distribution is somewhat rigid in these species. In our experiments with aphid milkers and aphid guards belonging to the same aphid tending group, when ants were experimentally forced to change their roles, much food was lost (Reznikova and Novgorodova, 1998a; Reznikova, 2007). In species with small colony sizes of about 100–300 workers such as *Temnothorax* species, specialists are no better at their jobs than generalists, and sometimes even perform worse. In addition, most of the work in the colony is not performed by the most efficient workers (Dornhaus, 2008). Moreover, strict specialization is disadvantageous for a colony’s annual reproduction and growth during slave raids (Jongepier and Foitzik, 2016). It is possible that in red wood ants, with their colony sizes up to million individuals, the effectiveness of deep professional specialization is connected with their sophisticated communication system based on transferring from scouts to foragers the exact messages about the coordinates of remote goals (Reznikova, 2017). We suggest that further study of the distribution of cognitive responsibilities within colonies of different sizes and levels of specialization in different ant species may help to revise our understanding of the benefits of colony organization.

## Author Contributions

II conducted the experiments with insects. II and ZR discussed all problems and wrote the manuscript. ZR took part in the planning and conducting the experiments.

## Conflict of Interest Statement

The authors declare that the research was conducted in the absence of any commercial or financial relationships that could be construed as a potential conflict of interest.
